# Pathological complete response in MMR-deficient/MSI-high and KRAS-mutant patient with locally advanced rectal cancer after neoadjuvant chemoradiation with immunotherapy: A case report

**DOI:** 10.3389/fonc.2022.926480

**Published:** 2022-09-23

**Authors:** Mai Zhang, Hua Yang, Ling Chen, Kunli Du, Lina Zhao, Lichun Wei

**Affiliations:** ^1^ Department of Radiation Oncology, The First Affiliated Hospital, Air Force Medical University, Xi an, China; ^2^ Department of Pathology, The First Affiliated Hospital, Air Force Medical University, Xi an, China; ^3^ Department of Gastrointestinal Surgery, The First Affiliated Hospital, Air Force Medical University, Xi an, China

**Keywords:** locally advanced rectal cancer, neoadjuvant chemoradiation, immunotherapy, pathological complete response, abscopal effect

## Abstract

To date, preoperative chemoradiation (CRT) is the standard of care for patients with locally advanced rectal cancer (LARC) regardless of status of mismatch repair. Immunotherapy showed promising results in the neoadjuvant treatment trials in patients with mismatch repair-deficient (dMMR) or high microsatellite instability (MSI-H) LARC. The efficacy of CRT plus programmed death 1 (PD-1) inhibitor in these patients with complex gene mutation remains unclear. Additionally, very few studies reported on whether such combination could induce abscopal effect. We report a case of dMMR and MSI-H LARC with KRAS mutation that achieved pathological complete response of primary lesion and liver metastases after neoadjuvant short-course radiotherapy followed by four cycles chemotherapy of XELOX plus PD-1 inhibitor tislelizumab and a subsequent total mesorectal excision. This case indicates that this combined treatment strategy has remarkable clinical response both in locoregional and distant diseases, which potentially leads to reduction in the risk of distant metastases and better locoregional control for this subgroup of population.

## Introduction

Preoperative chemoradiation (CRT) is the standard of care for patients with locally advanced rectal cancer (LARC). CRT before surgery could downstage tumors and increase local control rate, with a pCR rate of 30% in the RAPIDO trial. However, distant metastatic rate remains high, which was 20% at 3 years in the RAPIDO trial (1–3). The mismatch repair-deficient (dMMR) or high microsatellite instability (MSI-H) phenotype, accounting for approximately <10% of all rectal cancer, was considered to be sensitive to immunotherapy (4, 5). The NICHE trial showed a pathological complete response (pCR) rate of 60% in dMMR/MSI-H patients after surgery, with neoadjuvant immunotherapy of ipilimumab and nivolumab (6). The latter phase II trial investigated the efficacy of neoadjuvant programmed cell death protein 1 (PD-1) inhibitor with celecoxib and found that the pCR rate was 88% in dMMR/MSI-H colorectal cancer (7). These findings indicated that neoadjuvant immunotherapy could achieve a remarkable clinical response in downstaging tumors in dMMR/MSI-H rectal cancer. KRAS mutation occurred in approximately 40% of all colorectal cancer (8). For mCRC patients with KRAS mutations, the standard treatment is chemotherapy with or without bevacizumab. Meanwhile, chemoradiotherapy followed by surgery is the standard of care for patients with LARC irrespective of KRAS status. A previous study has revealed that MSI-H/dMMR metastatic colorectal cancer (mCRC) with KRAS or NRAS mutations could not benefit from single PD-1 inhibitor compared with chemotherapy in overall survival (9). Furthermore, whether the occurrence of locoregional recurrence and distant metastases could decrease with neoadjuvant immunotherapy remains unclear due to the immaturity of the data.

The clinical efficacy of CRT plus PD-1 inhibitor in dMMR/MSI-H LARC with KRAS mutation was rare, especially whether such combination could induce abscopal effect. Here, we present a LARC case with dMMR/MSI-H status and KRAS mutation who received short-course radiotherapy, followed by four cycles chemotherapy of XELOX plus PD-1 inhibitor tislelizumab and subsequently by a total mesorectal excision with pCR in primary tumor. Interestingly, during the treatment, a lesion was noted in the liver by imaging, and metastasis was rendered clinically; however, no tumor cells were identified by biopsy and pathological evaluation. To the best of our knowledge, this phenomenon has not been reported.

## Case report

**Figure 1:**
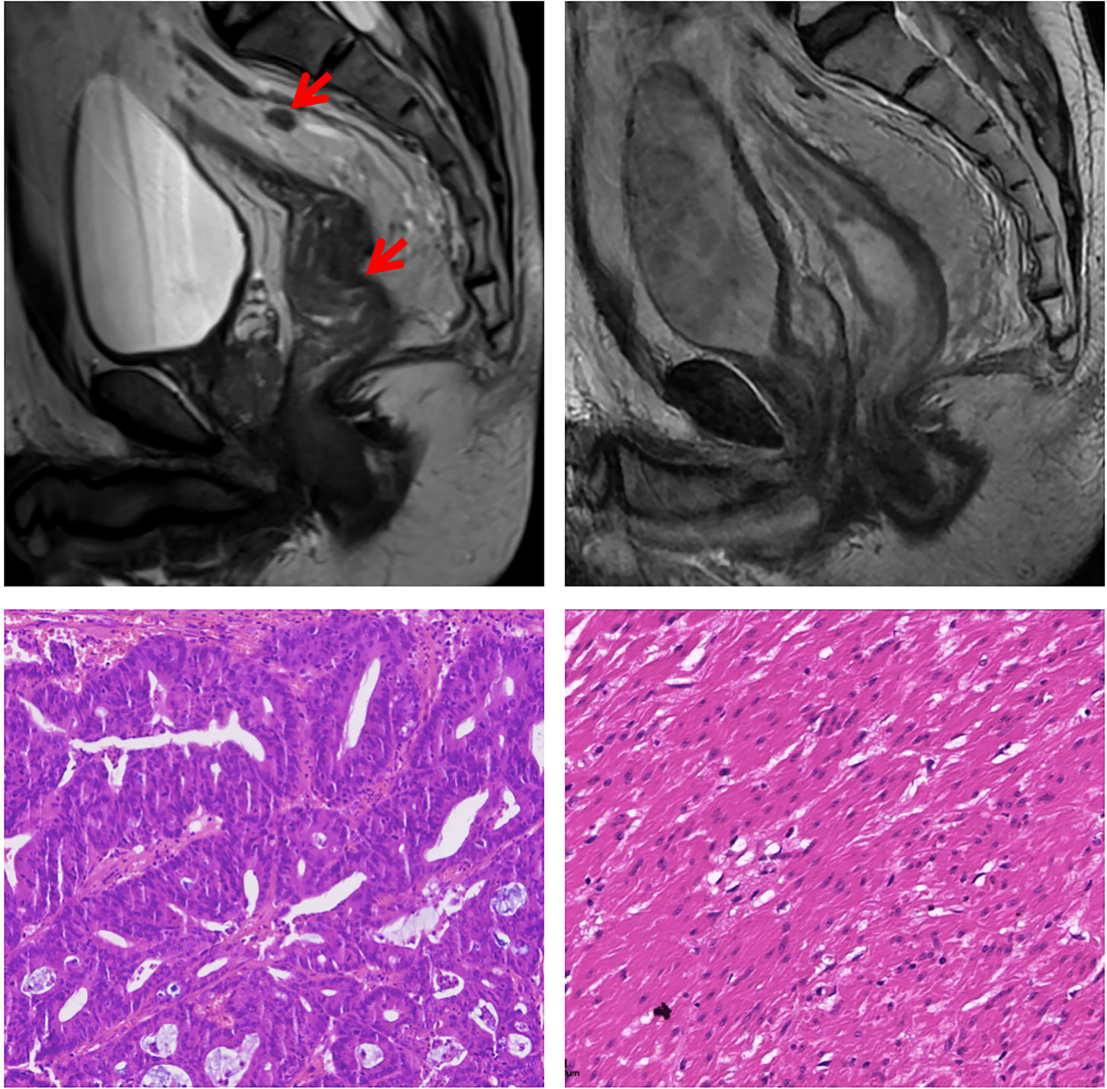
Magnetic resonance imaging and histological findings before and after treatment. In the upper row, Magnetic resonance imaging-T2 scans at diagnosis and before surgery showed partial response of the tumor. In the lower row, representative sections of tumor specimens before neoadjuvant treatment (left) and after the treatment (right) (hematoxylin and eosin staining × 200). This patient had pathological complete response of the primary tumor.

**Figure 2:**
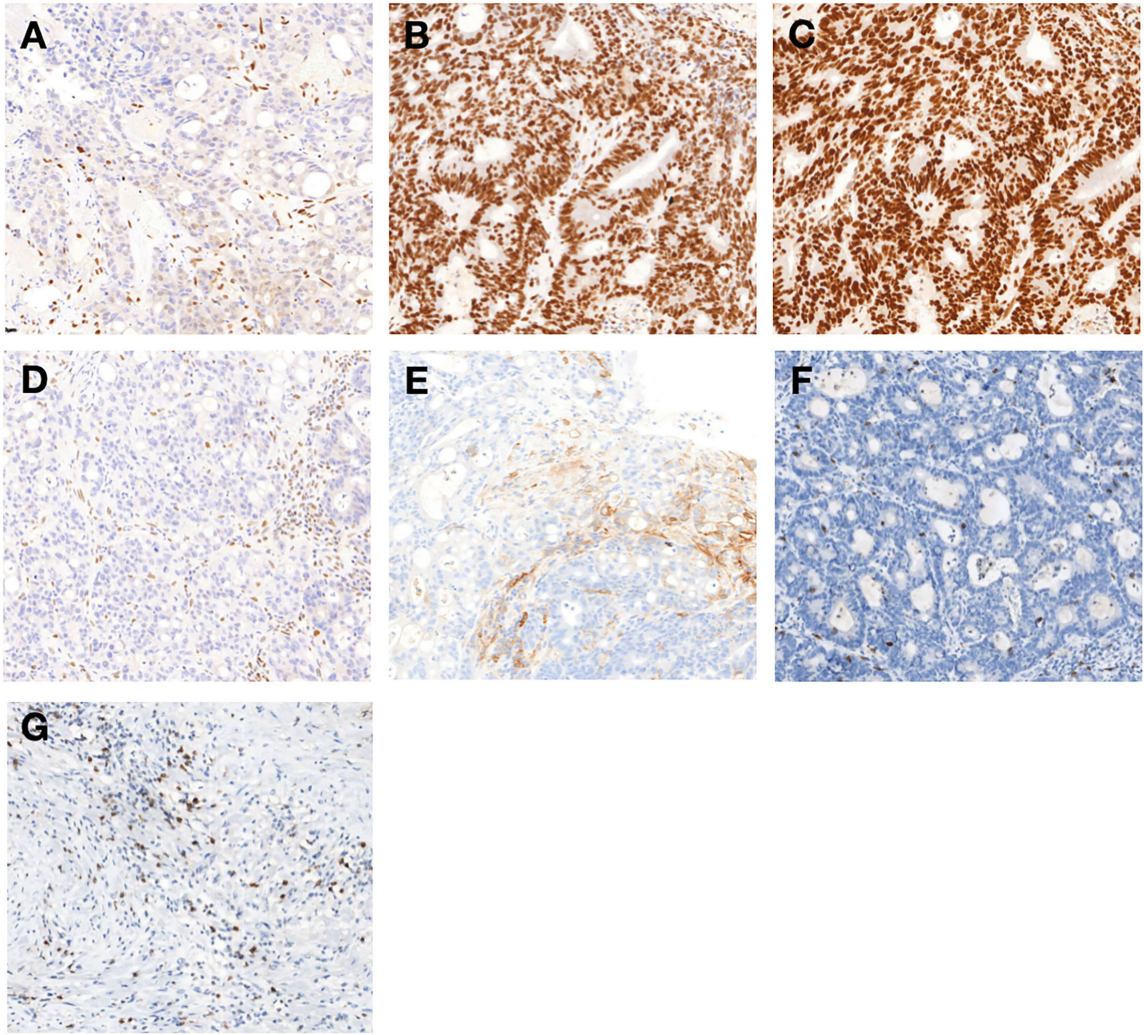
Immunohistochemical findings of primary tumor **(A–H)**: Immunohistochemistry of the protein MLH1 **(A)**, MSH2 **(B)**, MSH6 **(C)**, and PMS2 **(D)**. MSH2 and MSH6 were positively expressed, whereas MLH1 and PMS2 were lacking (200×). **(E)** The combined positive score of programmed cell death protein ligand 1 (PD-L1) expression in rectal tumor before treatment was grade 2 (200×). Immunohistochemical analysis of the rectal tumor microenvironment before treatment **(F)** and the tumor microenvironment was enriched with CD8+ T cells in primary tumor **(G)** after completion of treatment (200×).

A 53-year-old man was diagnosed as having locally advanced rectal cancer in April 2021, which was 5.5 cm to the anal verge and staged as cT3N2 by magnetic resonance imaging (MRI) (**Figure 1**). Mesorectal fascia (MRF) was negative, and extramural vascular invasion (EMVI) was categorized as grade 3. Histopathologically, it was a moderately to poorly differentiated adenocarcinoma. The immunohistochemistry for protein of mismatch repair gene showed a deficiency of MLH1 and PMS2, which meant that it was mismatch repair gene deficient (dMMR) (**Figures 2A–D**). Multiplex PCR-microsatellite analysis also confirmed the MSI-H status in all markers, and the combined positive score of programmed cell death protein ligand 1 expression was grade 2 (**Figure 2E**). A next-generation sequencing (NGS) panel spanning 952 cancer-related genes was performed, and FBXW7, ERBB2, ERBB3, MLH1, APC, CDK12, PIK3CA, and KRAS mutations were observed. The tumor mutation burden was 52.94 mutations/megabase (the methods and the identified mutations can be seen in the **Supplementary Material**).

Therefore, the tumor was classified as MSI-H/dMMR rectal cancer. No evidence of liver or other distant metastases was found at initial diagnosis through contrast-enhanced computed tomography of the chest and abdomen and abdominal ultrasound examination. Reported data of LARC showed a higher likelihood of pCR rate with neoadjuvant immunotherapy in patients with dMMR/MSI-H tumor. Owing to the patient’s persistent request for immunotherapy, and his dMMR/MSI-H LARC, we therefore decided within our multidisciplinary team to apply combined treatment strategy with PD-1 inhibitor tislelizumab and neoadjuvant chemoradiotherapy to enhance the efficacy of the anti-tumor therapy for his KRAS mutation. Short-course radiotherapy (5x5Gy) was performed at the beginning, and the patient then underwent four cycles of XELOX (capecitabine and oxaliplatin) with tislelizumab. Four weeks after completion of the treatment, restaging MRI showed a partial response of the rectal lesion (**Figure 1**). Biopsy pathology of the rectal lesion was negative.

**Figure 3:**
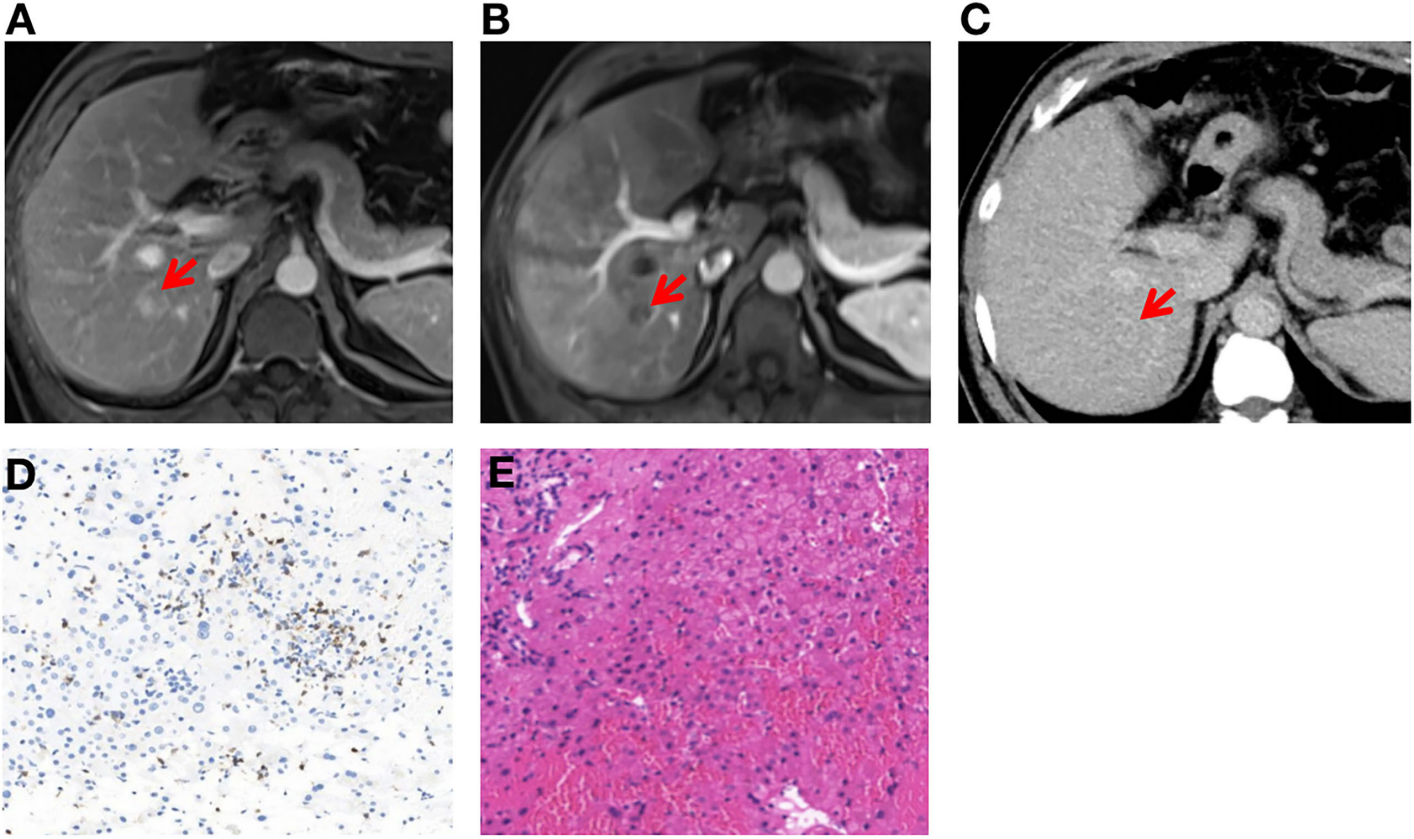
Clinical diagnosis and histological results of liver metastases **(A–E)**. **(A, B)** Dynamic-enhanced magnetic resonance imaging shows liver metastasis after neoadjuvant treatment and confirmed by contrast-enhanced ultrasonography. **(C)** Contrast-enhanced computed tomography shows no definite liver metastases before neoadjuvant treatment. **(D)** The tumor microenvironment was enriched with CD8+ T cells in liver metastases after completion of treatment (200×). **(E)** Post-treatment liver biopsy shows hepatic lobular structure with normal architecture, and no tumor cells were observed (200×).

Unfortunately, a liver lesion was detected on abdominal ultrasound examination and then clinically diagnosed as liver metastases (M1a) by dynamic-enhanced MRI and contrast-enhanced ultrasonography within our multidisciplinary team. The lesion size was 1.5 cm × 1.3 cm in MRI (**Figures 3A, B** **)**. A reexamination of the baseline computed tomography of abdomen was performed and a nearly invisible, obscure lesion was detected at the corresponding location, which could represent the early stage of metastases (classified as suspicious metastases—Mx) (**Figure 3C**). Examination of fine-needle aspiration biopsy showed hepatic lobular structure with many lymphocyte infiltration, especially CD8-positive T cells (**Figure 3D**). No tumor cells were observed (**Figure 3E**).

**Figure 4:**
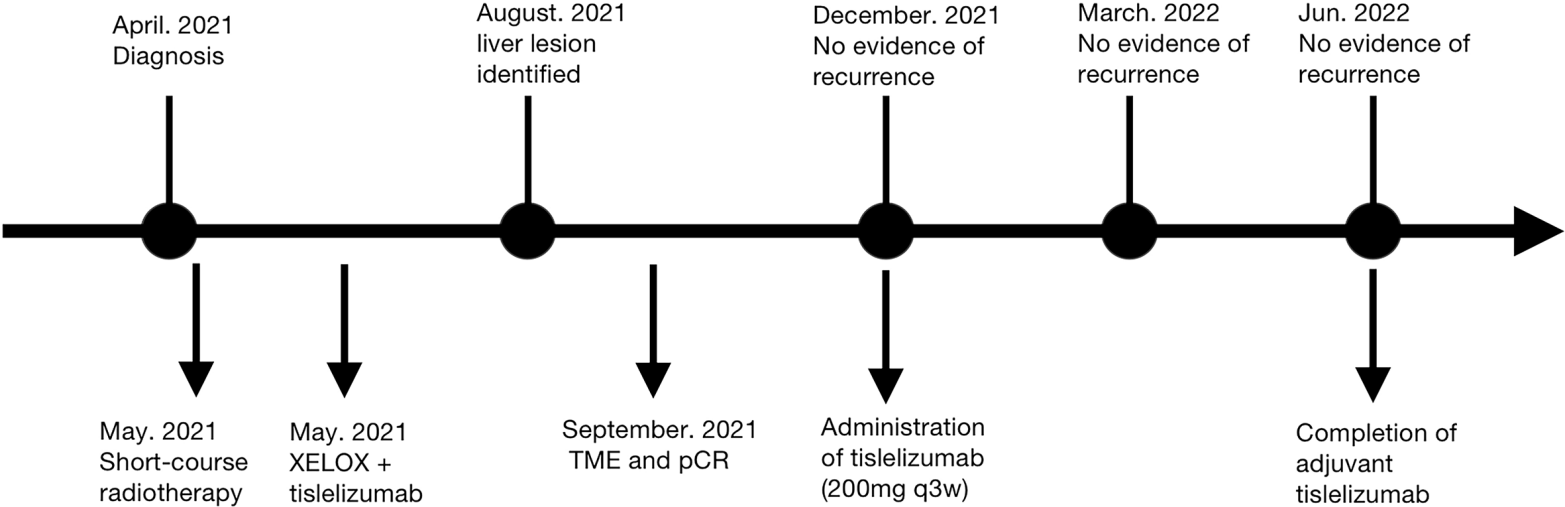
Timeline of disease status and corresponding treatment regimens. XELOX, capecitabine and oxaliplatin; TME, total mesorectal excision; pCR, pathological complete response.

We speculated that the possible reason of the new lesion in the liver was immunotherapy-related pseudoprogression. Meanwhile, radiofrequency ablation was delivered to the liver lesion for security purposes. One week later, total mesorectal excision was performed, the pathological result showed a complete response (**Figure 1**). Immunohistochemical analysis of the rectal tumor tissue after surgery showed that the tumor microenvironment was enriched with CD8+ T cells (**Figures 2F, G** **)**. The timeline of this case is shown in **Figure 4**.

PD-1 inhibitor plus chemoradiation was tolerable in this patient, without significant toxicities except for grade 2 thyroid-stimulating hormone increase. The surgery was delivered as scheduled without delay. Owing to the revised clinical stage, our multidisciplinary team planned to deliver adjuvant immunotherapy with single-agent tislelizumab for 6 months. The patient had finished adjuvant treatment. By the time of writing, this case has been followed up for 11 months and no evidence of disease recurrence was observed through regular re-examination (chest, abdominal, and pelvic computed tomography every 3 months, MRI of the rectum every 3 months, and CEA every 3 months).

## Discussion

Increasing data on immunotherapy is challenging standard preoperative therapy of LARC patients with dMMR/MSI-H (6, 7, 10). The care regime could become even more complicated when KRAS mutation was identified in these patients. The experience in this case suggests that the novel combined strategy of CRT with immunotherapy could significantly downstage tumors. Meanwhile, it had the capacity to control distant metastases. There will be a significant trend for decision-making of neoadjuvant treatment in LARC stratified by mismatch repair or microsatellite instability and KRAS status.

A recent publication showed that single-agent PD-1 inhibitor has achieved remarkable efficacy in 12 LARC patients with dMMR/MSI-H. After neoadjuvant treatment with dostarlimab for 6 months, all patients in the trial had a clinical complete response (cCR), without subsequent chemoradiotherapy or surgery (10). However, the sustainability of disease remission needs to be verified *via* the follow-up. Regrettably, KRAS status and its potential implications for immunotherapy were not mentioned in the article. In the KEYNOTE-177 trial, which compared pembrolizumab versus chemotherapy dMMR/MSI-H metastatic colorectal cancer (mCRC), the forest plot of subgroup analysis revealed that no significant difference was seen in the survival benefit between PD-1 inhibitor versus chemotherapy in patients with KRAS or NRAS mutations (HR 0.92 [95% CI 0.48–1.75]) (9). The CheckMate 142 study showed that dMMR/MSI-H mCRC patients with KRAS mutation might benefit more from dual immunotherapy (11). In a pooled retrospective analysis of non-small cell lung cancer (NSCLC) patients (n = 1,430), NSCLC patients with KRAS mutation derived the greatest benefit from chemotherapy plus immunotherapy compared with either immunotherapy alone or chemotherapy alone (12). It could be seen that single PD-1 inhibitor might be insufficient, and a combined treatment would be needed for dMMR/MSI-H mCRC with KRAS mutation. In the future, it would be worth comparing the efficacy and long-term survival benefit of combined immunotherapy such as CRT plus PD-1 blockade versus single-agent immunotherapy, especially for patients with complicated gene status.

For patients with LARC after neoadjuvant therapy, organ preservation was an alternative to operation in patients who achieved cCR. Because the patient did not achieve cCR, organ preservation and subsequent watch-and-wait strategy might not be appropriate for this patient. Whether such combined treatment could improve the cCR rate and lead to subsequent organ preservation in patients with KRAS mutation still warrant further investigation. Besides, the relationship between clinical and pathological response was not equivalent. In patients who achieved cCR after neoadjuvant treatments, 74.3% of the patients were identified as pCR with local excision as reported (13). Although it still needs to be refined, such as consensus criteria of clinical assessment of cCR and management of tumor regrowth, the wait-and-see strategy still provided another option for LARC.

Radiation could remodulate the tumor microenvironment including the distant sites and enhance immune-cell recognition and antigen release, which might lead to abscopal effect (14). The post-treatment specimen contained enriched CD8 + T cells, which was consistent with change in the immune microenvironment resulting from chemoradiotherapy. Neo-adjuvant CRT has been shown to upregulate PD-L1 expression in rectal cancer (15). The clinical efficacy of such combination had also been confirmed. Luke et al. found that stereotactic body radiation therapy to metastatic lesions followed by pembrolizumab resulted in an abscopal response rate of 26.9% in a single-arm trial (16). Welsh et al. found that 42% of patients with lung metastases had abscopal responses in non-irradiated lesions treated with ipilimumab and sequential radiotherapy (17). Theelen et al. confirmed abscopal effect in metastatic NSCLC with a statistically significant difference. The incidence of abscopal response rate of radiotherapy with PD-1 inhibitor was significantly higher than that of single-agent PD-1 inhibitor (41.7% *vs*. 19.7%, p=0.0039) (18). Therefore, immunotherapy could synergize with CRT and had a potential to reduce the risk of distant metastasis. The surprising observation of the liver lesion in this case was perhaps attributed to the abscopal effect enhanced by CRT-combined PD-1 inhibitor. However, the optimal pattern of dose fraction to efficiently induce an anti-tumor immune response or abscopal effect in rectal cancer remains ambiguous, and hypo-fractionated radiotherapy might have a non­inferior therapeutic effect compared to conventional fractionated radiotherapy from the reported data (19, 20).

About 1% of patients with locally advanced rectal cancer will have distant metastasis during neoadjuvant treatment, which is a true progression (3). Different from true progression, pseudoprogression is an atypical response pattern of immunotherapy, observed in 10% of metastatic colorectal cancer patients with MSI-H/dMMR who received immunotherapy (21). Pseudoprogression is often followed by partial and complete responses and is considered to be associated with superior overall survival compared with real progression or stable disease (22). In this case, pseudoprogression of liver metastatic lesion was consistent with the pCR of the rectal lesion, demonstrating that the combined treatment had remarkable efficacy both in local and distant disease.

There were some limitations in this report. For example, the biopsy tissue from the liver did not represent the whole lesion sometimes. Second, follow-up duration was relatively short.

Collectively, CRT plus immunotherapy should be one option of neoadjuvant treatment for LARC patients with dMMR/MSI-H status and KRAS mutation. How to combine CRT with immunotherapy still warrants further investigation to improve the synergistic effect without significantly increased toxicity. Additional trials are underway.

## Data availability statement

The raw data supporting the conclusions of this article will be made available by the authors, without undue reservation.

## Ethics statement

This study was reviewed and approved by Ethics committee of Xijing Hospital. The patients/participants provided their written informed consent to participate in this study. Written informed consent was obtained from the individual(s) for the publication of any potentially identifiable images or data included in this article.

## Author contributions

MZ, HY, LC, KD, LZ and LW are the members of multidisciplinary team. HY and LC collected the pathological, biological, and clinical data. MZ and LW drafted the initial manuscript. LZ and other authors reviewed or revised the manuscript and approved the final version. The corresponding author had full access to all data and had final responsibility for the decision to submit for publication. All authors contributed to the article and approved the submitted version.

## Conflict of interest

The authors declare that the research was conducted in the absence of any commercial or financial relationships that could be construed as a potential conflict of interest.

## Publisher’s note

All claims expressed in this article are solely those of the authors and do not necessarily represent those of their affiliated organizations, or those of the publisher, the editors and the reviewers. Any product that may be evaluated in this article, or claim that may be made by its manufacturer, is not guaranteed or endorsed by the publisher.
