# Supplementary material for: Pathological complete response in MMR-deficient/MSI-high and KRAS-mutant patient with locally advanced rectal cancer after neoadjuvant chemoradiation with immunotherapy: A case report
Source: Front Oncol. 2022 Sep 23;12:926480. doi: 10.3389/fonc.2022.926480 (PMC9545900; doi:10.3389/fonc.2022.926480)
Supplement: Supplementary file 1 [file DataSheet_1.doc]

Methods

Whole-exome sequencing

Tumour and matched normal DNA were extracted using the QIAamp DNA FFPE Tissue Kit (56404, Qiagen, Hilden, Germany) from formalin-fixed paraffin-embedded (FFPE) tissues. Libraries were constructed by the SureSelectXT Human All Exon V6 kit (Cat. No. 5190–8864, Agilent Technologies, Santa Clara, CA), then the DNA libraries were constructed with KAPA Library Preparation Kit (Kapa Biosystems Inc., Wilmington, USA) and sequenced with next-generation sequencing. Genomic DNA was fragmented, end-repaired, adenylated at the 3' ends, end-connected, amplified, purified, and size-selected in the process of library construction, then was sequenced on the Illumina X10 platform (Illumina Inc., San Diego, CA, USA).

Somatic mutation and copy number variant (CNV) calling

Sequencing data underwent mutation analysis and human genome build hg19 was used as the reference genome. The sequenced reads were mapped to the hg19 by Burrows-Wheeler Aligner (BWA version 0.7.15, default parameters, BWA-MEM algorithm).

Somatic SNVs and InDels were identified via GATK MuTect2 (version 4.1, default parameters). Mutations were annotated with software ANNOVAR (version 2016-02-01, default parameters). Copy number variant (CNV) was analyzed with ONCOCNV (version 6.6, default parameters).

Criteria for TMB calculation

TMB was calculated by counting the number of somatic, coding mutations across a 1.2 megabase (Mb) region, with computational germline status filtering. The total number of mutations counted is divided by the size of the coding region of the targeted territory. And reporting the result as mutations/Mb.

Criteria to report mutations

Reported mutations include synonymous point mutation, nonsynonymous point mutation, insertion mutation, deletion mutation and copy number variation and structural rearrangement. Non-coding alterations were not counted.
